# Heterologous mRNA vaccine booster increases neutralization of SARS-CoV-2 Omicron BA.2 variant

**DOI:** 10.1038/s41392-022-01062-3

**Published:** 2022-07-19

**Authors:** Gen Li, Zhongcheng Zhou, Peng Du, Meixiao Zhan, Ning Li, Xinxin Xiong, Shengjun Tang, Miao Man, Daniel T. Baptista-Hon, Ligong Lu

**Affiliations:** 1 Guangzhou Women and Children’s Medical Center, Guangzhou Medical University, Guangzhou, China; 2 Zhuhai Precision Medicine Center, Zhuhai People’s Hospital, The First Affiliated Hospital of Faculty of Medicine, Macau University of Technology, Zhuhai, Guangdong China; 3 Department of Bioinformatics and AI, Guangzhou Laboratory, Guangzhou, China; 4 University Hospital and Center for Biomedicine and Innovations, Faculty of Medicine, Macau University of Science and Technology, Taipa, 999078 Macau China; 5 Present Address: University Hospital and Center for Biomedicine and Innovations, Faculty of Medicine, Macau University of Science and Technology, Taipa, 999078 Macau China

**Keywords:** Vaccines, Preclinical research

**Dear Editor**,

Emerging epidemiology data indicate that the Omicron BA.2 sublineage is expected to become the dominant strain owing to its enhanced transmissibility.^1^ The BA.1 sublineage reduced the efficacy of neutralizing antibodies.^2,3^ The BA.2 sublineage may possess similar neutralizing antibody evasion.^3^ However, booster (3^rd^ dose) vaccination strategies, or three exposures to the SARS-CoV2 spike protein through natural infection elicits strong neutralizing antibody responses. This raises the question of whether booster doses also elicit neutralizing antibody responses against BA.2. We present in this study the neutralization activity of serum from human recipients of homologous booster (three doses of CoronaVac inactivated vaccine) or heterologous booster (inactivated vaccine priming dose with BnT162b2 third dose) vaccine regimens against pseudovirus containing the Omicron BA.2 spike protein. We also evaluated the neutralizing activities of 6 monoclonal antibodies, and serum from monkeys vaccinated with a recombinant RBD protein vaccine. Finally, we also evaluated cell entry mediated by the Omicron BA.2 spike protein.

The Omicron BA.2 contains over 30 mutations in the spike protein, with more than 10 of these located in the receptor binding domain (RBD). The locations of these mutations are highlighted in Fig. 1a, together with the D614G and the ancestral wild-type (WT) variant. Accordingly, we constructed luciferase-expressing pseudoviruses containing the WT, D614G and BA.2 spike proteins. We validated the function of these pseudoviruses by assessing their ability to infect HEK-293T cells stably expressing ACE2 and TMPRSS2 (hereafter referred to as HEK-293T-AT). Infection was quantified as an increase in luciferase activity (Fig. 1b). We found that HEK-293T-AT luminescence was higher following infection by D614G and BA.2 pseudoviruses, versus WT. Interestingly, luminescence was also higher in D614G pseudovirus infected cells than BA.2. Our data therefore indicate that the BA.2 spike protein is less able to mediate cell entry via ACE2 and TMPRSS2. This is consistent with the observation that cellular entry by Omicron variants have a reduced requirement for the ACE2/ TMPRSS2 pathway.^4^

**Fig. 1:**
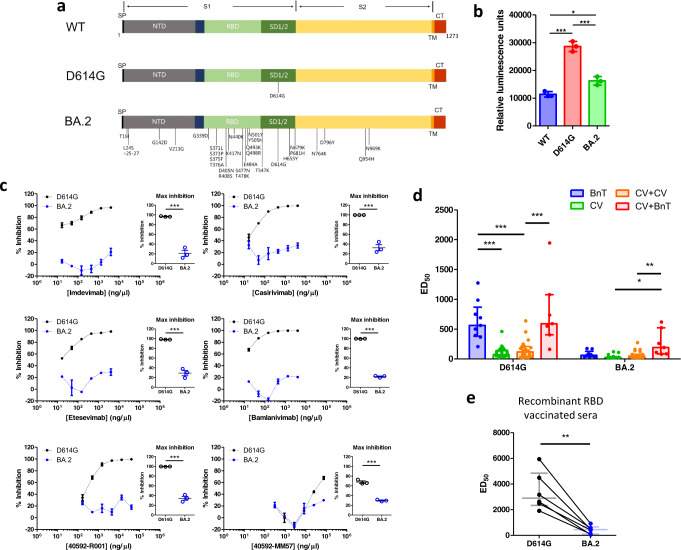
**a** Topological schematic of the SARS-CoV-2 spike protein, highlighting the locations of the mutations, relative to the ancestral WT strain. The spike protein is divided into the S1 and S2 (yellow) regions. The S1 contains the signal peptide (SP), the N-terminal domain (NTD; gray), the receptor binding domain (RBD; light green) and the subdomains 1 & 2 (SD1/2; dark green). Also shown are the transmembrane domain (TM; orange) and the cytoplasmic tail (CT; red). **b** Mean luminescence recorded from HEK-293T cells stably expressing ACE2 and TMPRSS2 following infection by pseudoviruses containing different spike proteins. Statistical analysis was performed using a one-way ANOVA (*P* < 0.0001). *Post-hoc* pairwise comparisons were performed using the Bonferroni correction (*P < 0.05; ***P < 0.0001). **c** Neutralizing activity for six monoclonal antibodies against D614G (black) and BA.2 (blue) pseudoviruses. Concentration-inhibition relationships of each monoclonal antibody are shown. Individual data points were joined with a straight line. No sigmoidal function was fitted to the data. Maximum inhibition of D614G or BA.2 pseudovirus entry into HEK-293T-AT cells were compared using a t-test (inset scatter plots). Data which significantly differed from each other are indicated using asterisks (***P < 0.0001). **d** Bar graph showing median [IQR] of ED_50_ from recipients of different vaccination regimens (BnT: two-dose BnT162b2, CV: two-dose CoronaVac, CV + CV: two-dose CoronaVac + CoronaVac booster, CV + BnT: two-dose CoronaVac + BnT162b2 booster). The data was compared with a two-way ANOVA. We find significant differences in the variant factor (*P* < 0.0001), vaccine factor (*P* < 0.0001) as well as significant interaction (*P* < 0.0001). We compared pairwise differences between different vaccination regimens using a Bonferroni *post-hoc* analysis. Data which significantly differ from each other are indicated using asterisks (*P < 0.05; **P < 0.001, ***P < 0.0001) **e** ED_50_ changes in neutralization activity on D614G and BA.2 pseudoviruses of sera from monkeys immunized with a RBD recombinant protein vaccine. Horizontal line and error bars indicate median and IQR, respectively. The data were compared with a paired *t*-test (*P* < 0.002)

Emerging data suggest that the BA.2 sublineage escapes the neutralizing activity of therapeutic monoclonal antibodies.^5^ We therefore assessed the neutralization activity of six different monoclonal antibodies, four of which are in clinical use (Supplementary Materials and Methods). Using the same infectivity assay as above, we incubated HEK-293T-AT cells with pseudoviruses in the absence or presence of increasing concentrations of antibodies. We quantified the effect of monoclonal antibodies using percentage inhibition (relative to the absence of antibodies). The concentration-inhibition effects are shown in Fig. 1c. The D614G pseudovirus was sensitive to neutralization by all monoclonal antibodies tested. By contrast, the BA.2 spike protein was insensitive to neutralization. Indeed, comparison of the maximum inhibition obtained for each monoclonal antibody revealed a significant reduction in inhibitory activity against the BA.2 spike protein (Fig. 1c inset scatter plots).

Neutralization antibody titers as a result of vaccination is reduced against the Omicron variant, but booster vaccinations can increase neutralizing antibody titers against the Omicron BA.1 and BA.2 sublineages.^3^ Indeed, the choice of booster vaccine matters, with mRNA-based vaccines producing higher levels of neutralizing antibodies.^6^ We therefore compared the neutralizing activity of different vaccination regimens involving the BnT162b2 vaccine (mRNA technology) and the CoronaVac vaccine (inactivated virus technology). We isolated serum from consented participants who have been vaccinated with either two-dose BnT162b2, two-dose CoronaVac, and also those who have taken either a CoronaVac or BnT162b2 booster following two CoronaVac priming doses. The recruitment details and the basic characteristics of the participants are shown in Supplementary Materials and Methods and Supplementary Table S1, respectively. Blood samples were taken on average 14 days after vaccination, and participants in the booster groups received their third dose 3 to 6 months following the priming doses. We used our HEK-293T based infectivity assay to evaluate the 50% effective dilution (ED_50_) of vaccinated sera. Overall, there was a reduction in the neutralizing activity against the Omicron BA.2 sublineage versus the D614G (Supplementary Fig. S1). We compared the different vaccination regimens using a two-way ANOVA (Fig. 1d). There was a statistically significant effect in the vaccination regimen factor, variant factor, and also significant interaction between the two factors. This complicated our interpretation of the results and we therefore limited our *post-hoc* analyses to a systematic pairwise comparison between the different vaccination regimens. For the D614G pseudoviruses, sera from two-dose BnT162b2 vaccinated individuals produced robust neutralizing activity. Sera from two-dose or three-dose CoronaVac recipients showed significantly reduced neutralization activity. Importantly, a BnT162b2 booster in the background of a CoronaVac priming dose produced neutralizing activity similar to two-dose BnT162b2. For the Omicron BA.2 pseudoviruses, two-dose BnT162b2, two-dose CoronaVac and three-dose CoronaVac all showed low neutralization activity (Fig. 1d), with some two-dose CoronaVac vaccination recipients having undetectable neutralizing activity. These were arbitrarily assigned an ED_50_ of 25 (the minimum fold dilution in this assay). Interestingly, a BnT162b2 booster in the background of two CoronaVac priming doses was able to significantly increase neutralization activity versus a CoronaVac booster and also two-dose CoronaVac. Our analysis therefore suggest that the Omicron BA.2 variant may completely escape neutralization in some CoronaVac recipients, but a heterologous vaccination booster with BnT162b2 can significantly increase neutralization. We have powered our recruitment according to a beta value of 90% for a two-way ANOVA analysis, which required 7 subjects per vaccine regimen (Supplementary Materials and Methods). To achieve this, we had recruited a larger number of CoronaVac than BnT162b2 recipients, consistent with the local population vaccination pattern, with most people opting for the CoronaVac vaccine and booster. This imbalance in sample size may affect our statistical power. However, our results are supported by similar studies against the Omicron BA.1 sublineage.^2^ Our findings are also consistent with other studies suggesting a heterologous boosting regimen with an mRNA vaccine in the background of viral vectored vaccine primers produced higher neutralizing titers.^7^ Nevertheless, we encourage colleagues to perform further studies to evaluate the influence of different vaccine regimens, including adenovirus vectored vaccines (e.g., ChadOx1), other inactivated vaccines (e.g., SinoVac), and other mRNA vaccines (e.g., Moderna) on their neutralization activity against different Omicron sublineages. This will help guide the best vaccination strategies.

The development of additional vaccines is important to bring the current pandemic under control, with recombinant spike protein fragments showing promise in recent clinical trials.^8^ We have previously developed and characterized the neutralization activity of a recombinant RBD vaccine in non-human primates, and reported high anti-RBD antibody levels and robust neutralization activities against SARS-CoV-2 WT and B.1.427/429 variant pseudoviruses.^9,10^ We vaccinated 6 Cynomolgus macaques (*Macaca fascicularis*) with the recombinant RBD vaccine (Supplementary Materials and Methods) and evaluated the ED_50_ neutralizing activity of their serum. Our data showed that the serum exhibited robust neutralizing activity against the D614G pseudovirus, but significantly reduced neutralization against the BA.2 pseudovirus (Fig. 1e). However, it should be highlighted that the ED_50_ was comparable to that of two-dose BnT162b2 against D614G (median [IQR] = 452 [97.8, 659]). These results suggest that recombinant RBD protein vaccines may confer enhanced protection against the Omicron BA.2.

Overall, we found that the BA.2 spike protein confers impaired cell entry, consistent with other observations that cell entry mediated by the Omicron VOC is impaired in the presence of TMPRSS2.^4^ The Omicron BA.2 spike protein mediated effective neutralization escape which can be mitigated through booster vaccinations, particularly with mRNA vaccines. Finally, our study also highlights the potential value of recombinant protein vaccines which showed robust neutralizing antibody titer against the Omicron BA.2 spike protein.

## Supplementary information

Supplementary Information

## Data Availability

The datasets generated during and/or analyzed during the current study are available from the corresponding authors on reasonable request.
