# Supplementary material for: Heterologous mRNA vaccine booster increases neutralization of SARS-CoV-2 Omicron BA.2 variant
Source: Signal Transduct Target Ther. 2022 Jul 19;7:243. doi: 10.1038/s41392-022-01062-3 (PMC9295091; doi:10.1038/s41392-022-01062-3)
Supplement: Supplementary file 1 — Supplementary Information [file 41392_2022_1062_MOESM1_ESM.docx]

Supplementary Materials for

Heterologous mRNA vaccine booster increases neutralization of SARS-CoV-2 Omicron BA.2 variant

Gen Li, Zhongcheng Zhou, Peng Du, Meixiao Zhan, Ning Li, Xinxin Xiong, Shengjun Tang, Miao Man, Daniel T. Baptista-Hon, Ligong Lu

Correspondence to: [dbaptistahon@must.edu.mo](mailto:dbaptistahon@must.edu.mo) or [lu_ligong@163.com](mailto:lu_ligong@163.com)

**This PDF file includes:**

Materials and Methods

Figure. S1

Table S1

Materials and Methods

Cell culture

HEK-293T cells (CL-00005; Procell, China) and HEK-293T cells stably expressing ACE2 and TMPRSS2 (CSC-ACE02; Creative Diagnostics, USA) were cultured in complete growth media containing 10% fetal bovine serum (FBS; PAN-Biotech, Germany) and 1% penicillin-streptomycin (Gibco, USA) in Dulbecco's Modified Eagle's Medium (DMEM, Thermo fisher, USA) in a humidified atmosphere at 37 °C with 5% CO_2_. Stable expression of ACE2 and TMPRSS2 were maintained with puromycin (0.5 µg/ml). Cells were routinely subcultured every 3-4 days. Transfection was performed using Lipofectamine 3000 (Invitrogen) according to manufacturer’s instructions

S-protein expression plasmids

cDNA encoding full length WT, D614G and Omicron BA.2 spike protein was subcloned into the pCAGGS vector (Genscript, Nanjing, China). All constructs used in this study were confirmed by Sanger sequencing.

Non-human primate serum

All procedures involved in the non-human primate study were reviewed and approved by the Institutional Animal Care and Use Committee of Institute of Sun Yat-sen University. Six adult (5-9 years old) Cynomolgus macaques (Macaca fascicularis) were used for the vaccine study, which were immunized with 20 μg RBD protein with Al(OH)3 plus topical imiquimod. The detailed methods for vaccine production were described previously^1,2^.

Human serum

The study protocol for recruiting and acquiring serum from immunized human subjects was approved by the University Hospital Clinical Research Ethics Committee of the Macau University of Science and Technology (Approval reference: UH/CREC/2022/01). Consented participants have completed either two-dose BnT162b2, two-dose CoronaVac, three-dose CoronaVac or two-dose CoronaVac with BnT162b2 booster.

We performed a power calculation to evaluate the number of participants required for this study using G-Power software. Another study investigating CoronaVac versus BnT162b2 vaccines found relatively large differences in neutralization activity against the Omicron BA.1 variant^3^. We therefore used a partial η2 value of 0.14 (corresponding to a large effect size) in our power calculations. Our calculations reveal to achieve a power of 90%, we will require a total sample size of 28 subjects, to detect statistically significant differences. This corresponds to 7 subjects per vaccination regimen. We recruited far more CoronaVac recipients than BnT162b2 recipients, consistent with the vaccination pattern in Macau. Therefore for this study, we recruited a total of 9 participants for two-dose BnT162b2, 23 for two-dose CoronaVac, 24 for three-dose CoronaVac and 7 for two-dose CoronaVac with BnT162b2 booster.

Whole blood was obtained by venipuncture and serum fraction obtained following centrifugation. For two-dose participants, samples were obtained at a median time of 14 days after the second vaccination. For three-dose participants, samples were obtained at a median time of 14 days after the booster vaccination, between 3 to 6 months after the second dose. Participant information is summarized in Supplementary Table S1.

Monoclonal antibodies

The following monoclonal antibodies were used in this study: Casirivimab-derived recombinant monoclonal mouse IgG2a (srbdc3-mab10, InvivoGen), Imdevimab-derived recombinant monoclonal mouse IgG2a (srbdc4-mab10, InvivoGen), Bamlanivimab-derived recombinant monoclonal mouse IgG2a (srbdc5-mab10, InvivoGen), Etesevimab-derived recombinant monoclonal mouse IgG2a (srbdc6-mab10, InvivoGen), SARS-CoV-2 (2019-nCoV) Spike Neutralizing Antibodies (Rabbit Mab (40592-R001, SinoBiological) and Mouse Mab (40592-MM57, SinoBiological)).

Production and quantification of pseudotyped viruses

The production and quantification of pseudotyped viruses was performed as described previously^2^. Briefly, HEK-293T cells were seeded at a density of 5×10^6^ cells in a 100 mm dish and were transfected with 12 μg pLOVE-luciferase-EGFP plasmid, 6 μg psPAX2 and 2 μg Omicron BA.2 spike protein expression plasmids with Lipofectamine 3000 (Invitrogen). 48 hours after transfection, SARS-CoV-2 pseudotyped viruses were harvested and filtered through 0.45 μm filter.

Infectivity assay

HEK-293T cells stably expressing ACE2 and TMPRSS2 were seeded at a density of 1×10^4^ cells in 96-well plates. Recovered pseudotyped viruses (described above) were diluted to 8×10^4^ particles per 100 μl in DMEM medium and added to each well. Luciferase activity was measured after 60 hours from lysed cells in the presence of luciferase substrate. Luminescence signal was detected using LumiStation 1800 Luminescence Microplate Reader (Shanghai Flash Spectrum Biotechnology Co., Ltd, China).

Cell–cell fusion assay

HEK-293T cells were transfected with pCMV-EGFP alone or in combination with Omicron BA.2 spike protein expression plasmids and cultured in complete media for 48 hours. HEK-293T cells stably expressing ACE2 TMPRSS2 were seeded at a density of 5x10^5^ in 12-well plates and allowed to settle at 37℃ for 5 hours, following which transfected HEK-293T cells were added at a density of 1×10^5^ cells. After co-culture at 37℃ for 12 hours, cell fusion was evaluated under an inverted fluorescence microscope (Leica, DMIL LED). The following criteria was used for cell fusion: 1) Fused cells increased in size by at least two-fold, and 2) the intensity of fluorescence in the fused cell was reduced. Five fields were randomly selected in each well to count the number of fused and unfused cells. Relative fusion was quantified as the percentage of fused cells in the presence of the Omicron BA.2 spike protein relative to the D614G spike protein. All experiments were performed at least three times.

Neutralization assay

HEK-293T cells stably expressing ACE2 and TMPRSS2 were seeded at a density of 1x10^4^ in 96-well plates. Recovered pseudotyped viruses were incubated in the absence or presence of serum samples or monoclonal antibodies (at dilutions of 1:25, 50, 100, 200, 400, 800, 1600, 3200, 6400, 12800) for 1 hour at 37°C before being added to cells. Cells were then incubated at 37°C for 12 hours before the viruses were removed. Cells were then lysed in the presence of luciferase substrate after another 48 hours, and luminescence signals quantified as above. Dilution-neutralization curves were generated and the sample 50% effective dilution (ED50) was calculated.

Quantification and statistical analysis

GraphPad Prism 8 was used for plotting and statistical analysis. Luminescence, syncytia area and percentage inhibition were expressed as mean ± SEM. ED_50_ values were expressed as median ± interquartile range. Statistical analyses on the effect of pseudotyped viruses on luminescence and syncytia area were performed using one-way ANOVA. Corrections for post-hoc pairwise comparisons were with the Tukey method. Pairwise comparisons of monoclonal antibody percentage inhibition between D614G and BA.2 spike proteins were done using the t-test. Paired comparisons of ED_50_ of monkey sera on D614G and BA.2 were performed using the paired t-test. A two-way ANOVA was used to evaluate the effect of different vaccination regimen on the neutralization of D614G and Omicron BA.2 spike protein. A Bonferroni post-hoc test was used to evaluate pairwise differences between different vaccination regimen. P-values of less than 0.05 were considered to be statistically significant.

**References**

1. Yang, J. et al. A vaccine targeting the RBD of the S protein of SARS-CoV-2 induces protective immunity. Nature **586**, 572–577 (2020).
2. Zhou, Z. et al. Assessment of infectivity and the impact on neutralizing activity of immune sera of the COVID-19 variant, CAL.20C. Signal Transduct. Target. Ther. **6**, 285 (2021).
3. Cheng, S. M. S. et al. Neutralizing antibodies against the SARS-CoV-2 Omicron variant BA.1 following homologous and heterologous CoronaVac or BNT162b2 vaccination. Nat. Med. **28**, 1–4 (2022).


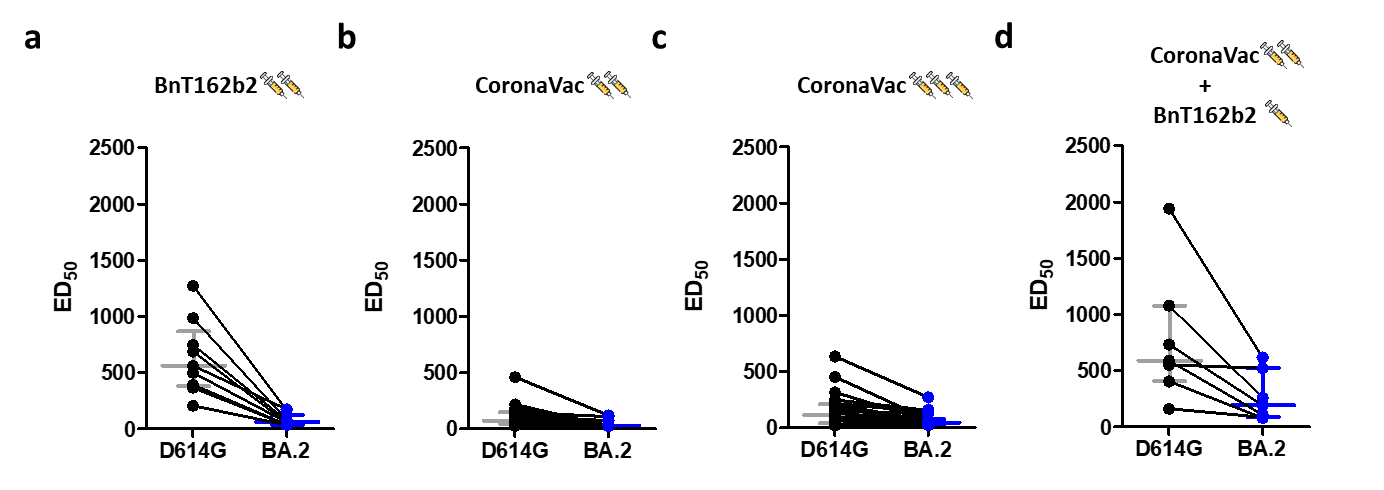


Figure. S1 Effect of immunized sera on SARS-CoV-2 spike protein variants. Neutralization activities of sera from recipients of (a) two-dose BNT162b2 (n = 9), (b) two-dose CoronaVac (n = 23), (c) homologous three-dose CoronaVac (n = 24) and (d) heterologous two-dose CoronaVac with BNT162b2 booster (n = 7) against D614G and BA.2 pseudoviruses. The fold-reduction against BA.2 pseudoviruses were 8-fold for two-dose BnT162b2, 2.1-fold for two-dose CoronaVac and 2.7-fold for BnT162b2 booster following CoronaVac priming doses. A number of samples in the two- or three-dose CoronaVac had undetectable neutralization at the lowest dilution tested. These were arbitrarily assigned an ED_50_ of 25. Horizontal line and error bars indicate median and IQR, respectively.

**Table S1. Vaccinated subject characteristics**

|  | **n** | **Age (median [range])** | **Number of days since last vaccine dose (median [range])** |
| --- | --- | --- | --- |
| **2-dose BNT162b2** | 9 | 32 [26-60] | 14 [14-14] |
| **2-dose CoronaVac** | 23 | 30 [22-37] | 14 [14-28] |
| **2-dose CoronaVac + CoronaVac booster** | 24 | 27.5 [22-47] | 14 [14-14] |
| **2-dose CoronaVac + BnT162b2 booster** | 7 | 54 [30-60] | 14 [14-28] |
